# The Maillard reaction in traditional method sparkling wine

**DOI:** 10.3389/fmicb.2022.979866

**Published:** 2022-08-26

**Authors:** Hannah M. Charnock, Gary J. Pickering, Belinda S. Kemp

**Affiliations:** ^1^Department of Biological Sciences, Faculty of Mathematics and Science, Brock University, St. Catharines, ON, Canada; ^2^Cool Climate Oenology and Viticulture Institute (CCOVI), Brock University, St. Catharines, ON, Canada; ^3^National Wine and Grape Industry Center, Charles Sturt University, Wagga Wagga, NSW, Australia; ^4^Sustainability Research Centre, University of the Sunshine Coast, Sippy Downs, QLD, Australia

**Keywords:** Maillard reaction, sparkling wine, aging, amino acids, sugars

## Abstract

The Maillard reaction between sugars and amino acids, peptides, or proteins generates a myriad of aroma compounds through complex and multi-step reaction pathways. While the Maillard has been primarily studied in the context of thermally processed foods, Maillard-associated products including thiazoles, furans, and pyrazines have been identified in aged sparkling wines, with associated bready, roasted, and caramel aromas. Sparkling wines produced in the bottle-fermented traditional method (*Méthode Champenoise*) have been the primary focus of studies related to Maillard-associated compounds in sparkling wine, and these wines undergo two sequential fermentations, with the second taking place in the final wine bottle. Due to the low temperature (15 ± 3°C) and low pH (pH 3–4) conditions during production and aging, we conclude that Maillard interactions may not proceed past intermediate stages. Physicochemical factors that affect the Maillard reaction are considered in the context of sparkling wine, particularly related to pH-dependent reaction pathways and existing literature pertaining to low temperature and/or low pH Maillard activity. A focus on the origins and composition of precursor species (amino acids and sugars) in sparkling wines is presented, as well as the potential role of metal ions in accelerating the Maillard reaction. Understanding the contributions of individual physicochemical factors to the Maillard reaction in sparkling wine enables a clearer understanding of reaction pathways and sensory outcomes. Advancements in analytical techniques for monitoring the Maillard reaction are also described, and important areas of future research on this topic are identified.

## Introduction

The Maillard reaction is a highly complex pathway, and has been studied across multiple disciplines including human physiology, pathology, geology, and flavor chemistry, in the 100+ years since its discovery in 1912 by the French chemist, Louis Camille Maillard (Maillard, 1912; Finot, 2005). The reaction is defined by the non-enzymatic condensation between the carbonyl group of a reducing sugar and the amine group of amino acids, peptides, or proteins, commencing in a multi-step reaction cascade *via* parallel and consecutive sub-reactions (Ames, 1990). Initially studied for its role in brown color formation and flavor contributions in thermally processed foods, Maillard chemistry is now understood to also occur in low-temperature conditions or during prolonged storage, and is even relevant *in vivo,* where it is implicated in human health and disease pathology (Ames, 1990; Kennedy and Knill, 2003; Nursten, 2005; Baynes and Gillery, 2014). In food science, the Maillard reaction is an established contributor to sensory, color, textural, and nutritional properties of foods, and also impacts protein functionality and digestibility (Hodge, 1953; Nursten, 2005; Baynes and Gillery, 2014; Lund and Ray, 2017). However, limited research has evaluated the formation of Maillard reaction-associated compounds under the unique physicochemical conditions relevant to sparkling wine production and aging. Sparkling wines are produced and aged in low temperatures (15 ± 3°C), with low pH (pH 3–4), pressure (6 atm), high acidity (titratable acidity 7–12 g/l), ethanol presence (12% *v/v*), and long-term aging on dead yeast *lees* (9 months to several years; Kemp et al., 2015). Maillard reaction-associated products including furans, acryl amides, and nitrogen, sulfur, and oxygen heterocycles, have been identified in aged sparkling wines (Cutzach et al., 1999, 2000; Marchand et al., 2000, 2011; Pripis-Nicolau et al., 2000; Keim et al., 2002; Silva Ferreira et al., 2003; Tominaga et al., 2003b; Jeandet et al., 2015; Le Menn et al., 2017). These compounds are reported to contribute roasted, bready, nutty and caramel aromas to the overall wine flavor and are believed to be produced *via* Maillard or Maillard-like activity during wine aging (Cutzach et al., 1999, 2000; Marchand et al., 2000, 2011; Pripis-Nicolau et al., 2000; Keim et al., 2002; Silva Ferreira et al., 2003; Tominaga et al., 2003b; Jeandet et al., 2015; Le Menn et al., 2017).

Despite progress over the past century, elucidating the mechanistic pathways leading to the formation of Maillard-associated products remains a fundamental challenge in the field due to the complexity of the reaction and quantity of reaction products. In sparkling wine, Maillard pathways are further convoluted by the complexity of the matrix, which contains hundreds of compounds from different chemical families. Additionally, the composition of Maillard-relevant precursors in sparkling wine can be impacted at multiple stages of the production process; for instance, from viticultural factors (grape variety, vineyard site, processing equipment), fermentation, yeast autolysis, and winemaking additions (sugar, nutrients, adjuvants). To the best of our knowledge, this review presents the first assessment of available literature on the formation of Maillard reaction-associated compounds in sparkling wine, including applicable information from other research in low-temperature and/or low pH conditions. Maillard reaction pathways are discussed in the context of sparkling wine, and factors that may contribute to the formation of associated compounds are evaluated with a focus on the composition and origins of precursor species during the production process.

## Sparkling wine production

Sparkling wine is produced globally and incorporates distinct production processes, which delineate the main categories of sparkling wines, comprising the forced infusion of carbon dioxide (CO_2_) into a still base wine (i.e., carbonation, associated with lower quality sparkling wines), the generation of CO_2_ during the second fermentation of a base wine in pressurized tanks (i.e., Charmat method), or by a second fermentation in the wine bottle (i.e., transfer method or in the traditional method/*Méthode Champenoise*; Culbert et al., 2017). In the transfer method, wines undergo a second fermentation in the bottle and are subsequently blended in a pressurized tank to remove the dead yeast *lees*. Traditional method wines remain in the bottle following second fermentation and aging, where yeast sediment is removed from individual bottles during a process called disgorging. Traditional method wines are increasingly popular on a global scale (Kemp et al., 2015), and are the primary focus of this review due to their established aging potential and representation in existing literature related to the development of Maillard reaction compounds. Examples include Champagne and Crémant d’Alsace from France, and Cava from Spain.

**Figure 1:**
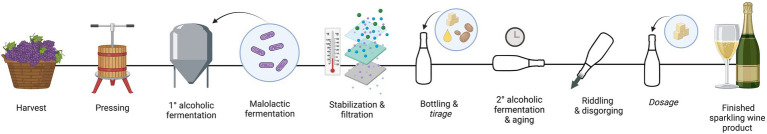
Method for traditional method sparkling wine production.

During the traditional method sparkling wine production process (Figure 1), harvested grapes are typically pressed as whole clusters to minimize phenolic extraction and sediment accumulation in the juice (Alexandre, 2019). An initial/primary alcoholic fermentation transforms the grape juice to a still base wine, and malolactic fermentation (MLF) may be combined or subsequently initiated. Lactic acid bacteria (typically *Oenococcus oeni*) carry out MLF, transforming malic acid into lactic acid for aroma and flavor enhancement, reduction of total acidity, and improved microbial stability (Virdis et al., 2021). Stabilization and filtration are often performed at this stage to prevent sediment and the formation of potassium or calcium tartrate crystals in the finished wine, which can cause wine gushing and product loss. Stabilization is typically achieved by storing wines at cold temperature (−4–2°C) to precipitate tartrates, although electrodialysis, ion-exchange resins, and the use of carboxymethylcellulose may be used as alternative strategies (Low et al., 2008; Gerbaud et al., 2010; Zoecklein et al., 2013). Clarification improves the clearness of the wine prior to bottling for the second alcoholic fermentation, and is most commonly achieved by filtration or crossflow filtration (Kemp et al., 2015). Subsequently, the second alcoholic fermentation takes place in the same bottle that is later purchased by the consumer. *Liqueur de tirage* (*tirage*), a combination of yeast, sugar, nutrients, and an adjuvant/riddling aid, is added to the wine to initiate this process. Throughout the second fermentation, CO_2_ gas is trapped and imparts the effervescent character to wine with post-fermentation pressures of approximately 6 atm (Alexandre, 2019).

During and following second fermentation, the wine is stored horizontally while in contact with the yeast cells (*lees* aging) for a period ranging from 9 months to several years. Yeast autolysis transforms and releases amino compounds including peptides, proteins, and enzymes, but also lipids and volatile aromatic compounds, into the wine, leading to distinct characteristics and sensory attributes. For example, select amino acids are aroma compound precursors, some peptides and proteins have sweet and bitter tastes and can influence foam stability, and mannoproteins contribute to the mouthfeel (body) of wine, etc. (Alexandre and Guilloux-Benatier, 2006). Following aging, riddling is carried out, where bottles are gradually tilted and rotated to accumulate autolyzed yeast and sediment in the bottle neck with aid of the adjuvant for compacting *lees*. This sediment is subsequently removed (disgorging) by freezing the neck of the bottle in glycol or a calcium chloride solution before quickly opening the crown cap, where pressure ejects the frozen deposit (Kemp et al., 2015). To account for lost volume, remaining wine is added, along with an optional final sugar addition, *liqueur d’expedition* (*dosage*), to adjust the target sweetness and balance of the finished wine. Additionally, sulfur dioxide (SO_2_) may be added at various stages throughout production, principally to base wine or in the *dosage* liqueur, to inhibit microbial spoilage and oxidation. Compounds including sugars, amino acids, proteins, and other matrix additives (e.g., SO_2_, adjuvants) that may be involved in the Maillard reaction thus are modified during production stages, altering potential reaction pathways and outcomes during bottle-fermented sparkling wine production and aging.

## Maillard reaction pathways

Due to the complexity of the Maillard reaction, hundreds to thousands of associated products may result from a reaction between a single sugar and amino acid (Ames, 1990; Golon et al., 2014). Disentangling the mechanistic steps of the Maillard reaction in sparkling wine is further convoluted by the diversity of potential reactants, leading to an increasingly large number of Maillard reaction-associated products. Generally, two reaction frameworks have been widely accepted for characterizing key stages of the Maillard reaction, as proposed by Hodge (1953) and more recently by Yaylayan (1997), and these are examined below in the context of sparkling wine.

### Hodge’s framework

**Figure 2:**
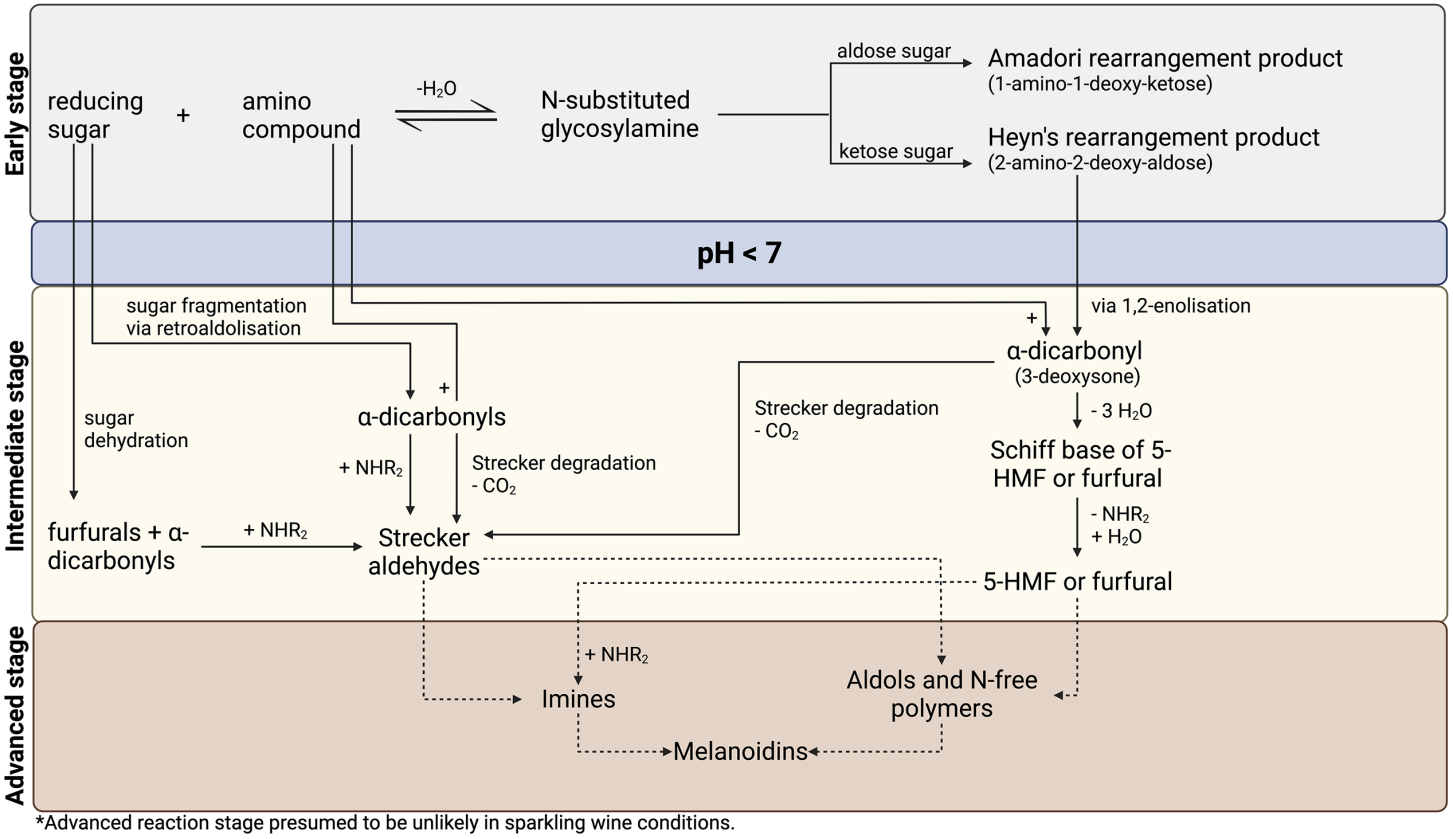
Proposed reaction pathway for the Maillard reaction in sparkling wine, adapted from Hodge (1953) and Nursten (2005).

In 1953, the first comprehensive scheme of the Maillard reaction was proposed by American scientist, Hodge (1953), and is still widely accepted in current literature. Hodge (1953) delineates three distinct reaction stages: early, intermediate, and advanced (Figure 2). In this reaction outline, pH-dependent steps are identified, therefore isolating pathways for low pH (<7) systems such as sparkling wine (pH 3–4). However, certain reaction stages are unlikely in sparkling wine. Specifically, in the advanced stage, brown pigments, known as melanoidins, and polymeric compounds are produced through the dehydration and polymerization of intermediate compounds (Nursten, 2005), with reported antioxidant, antimicrobial, and chemo preventive effects *in-vivo* (Hayase et al., 2006; Wang et al., 2011). However, since Maillard activity in sparkling wine proceeds under mild conditions due to the absence of heat, it is unlikely that advanced reaction products will be formed (Cutzach et al., 1999, 2000; Marchand et al., 2000, 2011; Silva Ferreira et al., 2003; Tominaga et al., 2003b; Le Menn et al., 2017).

Anticipated reaction pathways leading to the formation of Maillard reaction-associated products in sparkling wine conditions are identified and briefly described in this section. More recent advances in understanding Maillard chemistry, including sugar and amino acid degradation (Nursten, 2005), are also discussed. A consolidated reaction pathway for the Maillard reaction in sparkling wine, based on the model of Hodge (1953) with additional degradation pathways by Nursten (2005), is presented in Figure 2.

#### Early stage

In the early stage of the Maillard reaction (Figure 2; top section), a condensation reaction between the carbonyl group of a reducing sugar and the amino group of amino acids, peptides, or proteins forms an N-substituted glycosylamine, which reversibly dehydrates to form a Schiff base (Hodge, 1953; Nursten, 2005). Depending on the structure of the reducing sugar, this Schiff base irreversibly rearranges to form a stable Amadori rearrangement product (1-amino-1-deoxy-2-ketose) for aldose sugars, or a Heyns rearrangement product (2-amino-2-deoxy-aldose) for ketose sugars. Products of the reaction at this stage are reported to be colorless (Nursten, 2005).

#### Intermediate stage

During intermediate reaction stage (Figure 2; middle section), Amadori and Heyns rearrangement products can react *via* two routes in a pH-dependent manner, whereby they are converted into reactive α-dicarbonyl species that continue in the reaction cascade. In acidic (pH < 7) conditions relevant to sparkling wine (pH 3–4), a 1,2-enolization occurs *via* 3-deoxy-1,2-dicarbonyls, producing 3-deoxysone, an α-dicarbonyl (Nursten, 2005). In reactions involving glucose, the 3-deoxysone product is 3-deoxyglucosone and can be subsequently dehydrated to 5-hydroxymethyl-furfural (5-HMF; Hodge, 1953; Nursten, 2005; Nashalian, 2016), a commonly reported aging and quality marker in sparkling wine studies (Silva Ferreira et al., 2003; Serra-Cayuela et al., 2013a,b, 2014; Elcoroaristizabal et al., 2016). Conversely, in neutral or alkaline (pH > 7) conditions, the Amadori product proceeds by 2,3-enolization, leading to the formation of either 1-deoxysone or 4-deoxysone, generating reductones and other α-dicarbonyls. In each case, α-dicarbonyls rapidly react with nucleophiles such as the α-amino group of amino acids *via* Strecker degradation, wherein transamination produces an α-ketoacid, which is subsequently decarboxylated to a Strecker aldehyde with one less carbon than the corresponding amino acid (Hodge, 1953). Products of Strecker degradation are reported to range from colorless to yellow (Nursten, 2005), although measuring the formation of these browning pigments (absorbance at 420 nm) in white sparkling wine studies is convoluted by oxidative browning (Ibern-Gómez et al., 2000; Serra-Cayuela et al., 2013a, 2014). Therefore, browning measurements are a poor indicator of Maillard activity in white wines. Strecker aldehydes are important reducing compounds, which may react with each other and/or sugar-derived Maillard intermediates, thus re-initiating a portion of the reaction cascade (Nursten, 2005; Lund and Ray, 2017). Alternatively, they may be involved in the production of melanoidins and in advanced reactions (Nursten, 2005; Nashalian, 2016), although not relevant to sparkling wine conditions. The products from various phases of the intermediate reaction stage are of key importance to the sensory implications of the Maillard reaction, where aroma compounds with distinct bready, toasted, caramel, meaty, nutty, baked, and burnt odors are generated (Nursten, 2005; Cerny, 2008).

#### Amendments to early and intermediate stages of Hodge’s framework

Sugar dehydration and fragmentation (Figure 2; left side) are also important considerations in the Maillard reaction in the early and intermediate stages, as they occur under acidic conditions leading to the production of furfurals and other α-dicarbonyl species (Nursten, 2005; Lund and Ray, 2017). Nursten (2005) clearly outlined the importance of sugar dehydration and fragmentation to the overall reaction pathway. In acidic conditions (pH < 7), furfural compounds are produced by sugar dehydration, compared to reductone formation in neutral or alkaline conditions (pH > 7; Nursten, 2005). On the other hand, sugar fragmentation occurs *via* retroaldolization (decomposition into aldehyde or ketone plus carbonyl compound), producing a wide range of α-dicarbonyl compounds with varying reactivity and browning capacity (Nursten, 2005; Hemmler et al., 2018). In each case, these sugar breakdown products may go on as reactive intermediates in the Maillard reaction cascade (Nursten, 2005; Hemmler et al., 2017).

#### Advanced stage

In advanced reactions (Figure 2; lower section) typically involving high-heat, dehydration and polymerization of intermediate compounds and Strecker aldehydes lead to the formation of melanoidins and advanced glycation end products (AGEs; Nursten, 2005). Melanoidins are the primary contributors to browning and have a nitrogen-containing polymeric structure. They have been of particular interest in the food industry due to their effect on human health as well as their ability to alter product flavor, texture, and color (Wang et al., 2011). As previously discussed, the physiological conditions of sparkling wine render the formation of melanoidins and AGEs unlikely.

### Yaylayan’s alternative scheme

**Figure 3:**
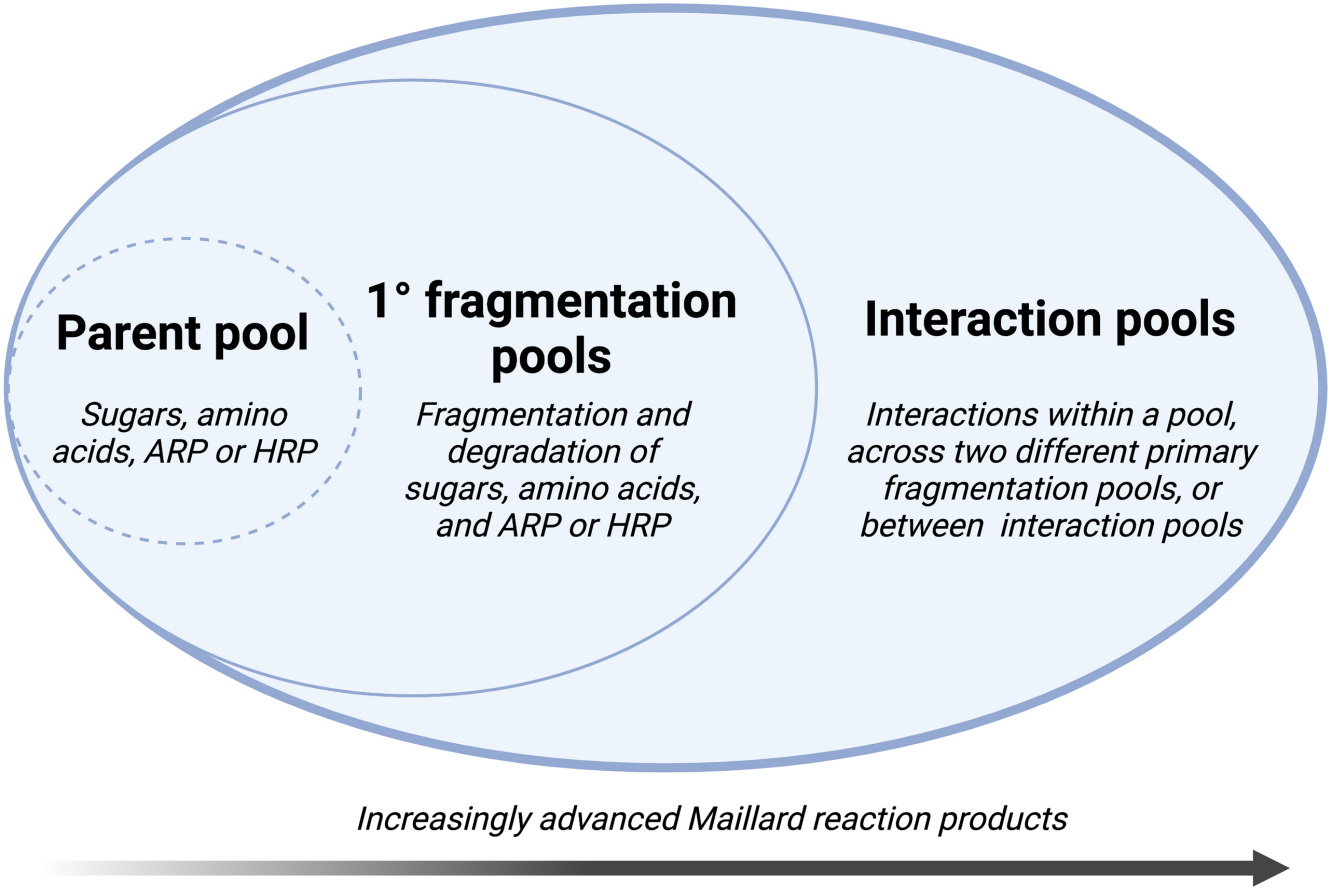
Conceptual framework for the Maillard reaction based on the formation and interaction of “chemical pools,” adapted from Yaylayan (1997) (ARP, Amadori rearrangement product; HRP, Heyns rearrangement product).

Although Hodge’s framework provides an important foundation for understanding the basic organic chemistry behind Maillard transformations, a broader overview has been proposed by Yaylayan (1997) (Figure 3). In this scheme, “chemical pools” are identified as building blocks for consecutive and parallel sub-reactions. A “parent pool” of fundamental precursors, including sugars, amino acids, and their immediate reaction products (Amadori or Heyns reaction products – 2-amino-1-deoxy-2-ketose for aldose sugars or 2-amino-2-deoxy-aldose for ketose sugars, respectively) determines the course of the reaction pathway. Subsequent interaction pools represent decomposition products from precursors, which may either exist as stable intermediates or react further with products from the same or other pools. In advanced reactions, heterocycles, high molecular weight products, and polymeric compounds are produced through interactions with and within increasingly complex interaction pools (Yaylayan, 1997). In a complex system such as sparkling wine, Yaylayan’s framework simplifies the reaction into discrete pools by linking the origins of reacting species and allowing for a more comprehensive view of these interactions (Yaylayan, 1997).

## Factors affecting the Maillard reaction

The rate and extent of the Maillard reaction relate to physiochemical conditions, including processing temperature, time, pH, composition (origin, concentration, and proportion of reactants), metal ion content, pressure, sulfur dioxide (SO_2_) content, and water activity (a_w_) (Ames, 1990; Ase et al., 1996; Laroque et al., 2008; Wong et al., 2015). A discussion of each factor and its relevance to sparkling wine are presented in the following sections.

### Temperature and time

In the context of food research, Maillard reaction literature primarily addresses thermally processed products. Although there have been significant advancements in Maillard reaction research over the last decade, comparatively few studies have evaluated foods with low-temperature processing and/or long-term aging such as honey (Brudzynski and Miotto, 2011), dairy products (Griffith and Hammond, 1989; Li et al., 2021), aged garlic (Ide et al., 2002), dried seeds (Sun and Leopold, 1995) and pasta (Anese et al., 1999), as well as various wine styles (Cutzach et al., 1999; Pripis-Nicolau et al., 2000; Marchand et al., 2002; Le Menn et al., 2017; Pereira et al., 2017).

Low temperatures significantly reduce the extent of Strecker degradation (Hodge, 1953), which is known to produce Strecker aldehydes – key odorants and contributors to browning in heated foods including bread, coffee, cocoa, beer, and meats (Rizzi, 1999; Vanderhaegen et al., 2006; Smit et al., 2009; Resconi et al., 2013). Despite reduced Strecker degradation in low-temperature systems, several Strecker degradation products (thiazole, 2-acetylthiazole, trimethyloxazole, 2-furanmethanethiol, and thiophene-2-thiol) have been identified in model wine conditions (pH 3.5, 12% *v/v* ethanol, 10–20°C) during reactions between α-dicarbonyl compounds (glyoxal, methylglyoxal, diacetyl, pentane-2,3-dione, acetoin, acetol, ethanal) and cysteine (Marchand et al., 2000). This suggests that although Strecker degradation is reduced, it is not inhibited in wine. Additionally, Marchand et al. (2000), determined that all five Strecker degradation compounds exhibited low (μg/L) sensory perception thresholds and odor activity values (OAVs; compound concentration divided by odor threshold in water) greater than 1, indicating their likely importance to sparkling wine flavor (Marchand et al., 2000).

Similarly, increased reaction temperatures are linked to a greater extent of the Maillard reaction, and thus high molecular weight product formation, although this is also dependent on the reaction duration (Ames et al., 2001; Martins and Van Boekel, 2005; Nursten, 2005; Rydberg et al., 2005). Indeed, the relationship between time and temperature is critical to the Maillard reaction, and this phenomenon was first reported by Maillard (1912). The implications of time and temperature on the sensory qualities of the reaction were later described by Reineccius (1990), who identified that the formation of Maillard products (and their respective aromas) will not be identical under altered time and temperature conditions. This has been repeatedly supported in the literature (Ames et al., 2001; Martins and Van Boekel, 2005; Nursten, 2005; Rydberg et al., 2005) and highlights the need for research on the Maillard reaction in wine to be carried out in representative wine-like temperature conditions.

### Role of pH

It has also been established that the Maillard reaction occurs more readily in alkaline than acidic conditions due to differences in the structural conformation of sugars and amino acids prior to the initial condensation interaction (Hodge, 1953; Martins and Van Boekel, 2005). For amino acids, the deprotonated version of the amino group is the reactive species, the proportion of which increases with pH (Ajandouz et al., 2001; Martins and Van Boekel, 2005). For sugars, the open-chain *keto* structure is the reactive species, and sugars can exist as many tautomeric forms in equilibrium depending on pH and temperature (Yaylayan et al., 1993; Martins and Van Boekel, 2005). In a model study of fructose by Yaylayan et al. (1993) (1.1–2.8 M solutions of *D*-fructose in deuterated water), acid–base catalysis was reported to have a minimal effect on the ring opening (pH 2–9), while temperature showed a significant effect (25–80°C). The authors reported that at room temperature (25°C), the composition of the reactive *keto* structure of fructose was only ~1% at pH 3 with the remaining 99% in tautomeric closed structure forms, and at pH 9, this ratio remains largely the same. Conversely, when the model solutions were held at pH 3 and heated to 80°C, the composition of the fructose *keto* structure increased to ~13%. In sparkling wines at pH 3–4, the reactive proportion of reducing sugars is therefore likely to be minimal, indicating that only a small percent of residual sugars would be accessible for Maillard activity under low pH and low temperature conditions. Additionally, the open-chain structure of glucose may also undergo a *keto*-*enol* tautomerism to fructose, although this occurs primarily in basic conditions and is therefore less pertinent to sparkling wine (Cremer and Eichner, 2000; Nashalian, 2016). Future research related to the structural conformation of sugars in sparkling wine conditions would be beneficial, particularly following *dosage* addition and subsequent aging. Further, the role of non-sugar carbonyl compounds (i.e., α-dicarbonyls) in the Maillard reaction requires further exploration under sparkling wine conditions.

Additionally, studies on Maillard reaction products in sparkling wine suggested that high organic acid content positively impacts the formation and stability of Maillard reaction products, which is favorable for sparkling wines, which contain an average titratable acidity of 7–12 g/l (Kemp et al., 2015).

### Reactant composition

The composition of Maillard reactants in sparkling wine is shaped by a multitude of factors, including grape variety, viticultural aspects, microbial populations, winemaking decisions, sugar additions, and the aging process (Moreno-Arribas et al., 1998; Martínez-Rodríguez et al., 2002; Hidalgo et al., 2004; Garde-Cerdán et al., 2011; Torresi et al., 2011; McMahon et al., 2017; Gutiérrez-Gamboa et al., 2018; Sartor et al., 2021). While the chemical composition of grapes differs by variety, other factors including viticultural management, climate, grape ripeness, vineyard location, rootstock, and soil conditions also impact juice and wine chemistry (Garde-Cerdán et al., 2011; Gutiérrez-Gamboa et al., 2018).

The Eurasian *Vitis vinifera* L. represents the majority of global wine grape cultivars (OIV, 2017), with upwards of 6,000 varieties in production (Maul and Toepfer, 2015). Non-traditional hybrid grape varieties including interspecific hybrid crosses between *V. vinifera* and *Vitis riparia* or *Vitis labrusca* are also used in sparkling wine production and are commonly grown in cold climate regions and/or areas with high disease pressure. However, the chemical composition of many non-*vinifera* grape varieties remain poorly characterized, thus limiting the development of novel varieties for sparkling wine production and our understanding of the Maillard reaction in non-*vinifera* wines. Sparkling wines produced from hybrid varieties are reported to be more susceptible to oxidation compared to *V. vinifera* varieties (Marks and Morris, 1993; Sartor et al., 2019), which may be associated differences in phenolic composition, particularly related to lower levels of flavonoids in some hybrid varieties which consequently decreases the wine’s capacity to resist oxidation (Singleton, 1987). Sartor et al. (2019) also identified increases in the phenolic compounds caffeic, gallic, and ellagic acids during aging of traditional method sparkling wines produced from *V. labrusca* hybrid varieties Villenave, Niagara, Manzoni and Goethe, with caffeic acid demonstrating a significant correlation to wine browning (*R* > 0.90). In a related study by the same authors, the amino acid composition of sparkling wines produced from *V. labrusca* hybrids was compared to Chardonnay sparkling wines over an 18-month period of *lees* aging, demonstrating that grape variety has a significant influence (*p* < 0.01) on the composition of all amino acids, except for asparagine and the ammonium ion (NH_4_^+^; Sartor et al., 2021). Additionally, the sum of amino acids was reportedly lower for all hybrid varieties compared to Chardonnay (Sartor et al., 2021). While this may suggest that hybrid grape varieties have a lesser potential for Maillard activity due to lower levels of amino acids, it is more likely that the concentration of specific reactive amino acids are a more reliable indicator of Maillard potential, although this has not yet been explored in the context of sparkling wine.

#### Sugar composition

The rate of sugar accumulation in the grape berries is highest between *véraison* and harvest, when the berry enlarges, changes color, and metabolizes acids as it approaches maturity. Sucrose is the primary sugar translocated from the vine to the fruit, although it is often hydrolyzed to fructose and glucose monomers by invertase enzymes. However, the ratio of glucose-fructose varies depending on the stage of berry development (Kliewer, 1967; Sabir et al., 2010). For example, glucose levels are higher than fructose in young berries of both table grapes and *V. vinifera* varieties, while fructose retention is slightly higher during *véraison* until maturity (Kliewer, 1967). Despite invertase converting sucrose into equal amounts of fructose and glucose, unequal sugar accumulation may be linked to the enzymatic conversion of glucose to fructose, different rates of metabolism, or the production of fructose from malic acid (Kliewer, 1967; Jackson, 1996b). Depending on grape species and cultivar., differences in residual sucrose levels are also evident, with approximately 2% sucrose in French American hybrids compared to 0.4% sucrose in *V. vinifera* (Jackson, 1996b), although *V. rotundifolia* (muscadine) wine grapes contain 19–37% sucrose (Basha et al., 2012).

Environmental factors in the vineyard have also been reported to influence grape sugar composition, with lower glucose-fructose ratios in warm seasons compared to cooler vintages (Kliewer, 1967; Tarko et al., 2014; Gashu et al., 2020). In the grape juice, glucose and fructose are consumed by yeast during primary fermentation, with glucose being preferentially metabolized (D’Amore et al., 1989). Prior to the second alcoholic fermentation, a specific amount of sugar is added in *tirage* (typically sucrose) based on the desired pressure and alcohol of the finished wine. At the end of alcoholic fermentation, small amounts (<0.1–2 g/l) of grape-derived sugars (apiose, raffinose, stachyose, fucose, melibiose, galactose, arabinose, xylose, rhamnose) and yeast-derived sugars (glucose, mannose) are not metabolized by yeast, and remain in the wine as monomers, or as subunits of larger polysaccharides (Jackson, 1996b; AWRI, 2016). Glucose and mannose levels arise during fermentation as the result of yeast-derived glucanase activity and yeast mannoprotein breakdown, respectively (Giménez et al., 2019). During aging, these unmetabolized sugars and polysaccharides are available for other potential chemical interactions. Additionally, glycosidically bound compounds in the wine may be liberated by enzymatic or acidic hydrolysis, thus further contributing to available sugar composition during aging (Ferreira and Lopez, 2019).

Several studies have characterized the monosaccharide components of polysaccharides as well as the evolution of polysaccharide families during both still and sparkling wine aging due to the effect of these compounds on fermentation, filtration, stabilization, foaming, and sensory characteristics (Moreno-Arribas et al., 2000; Nunez et al., 2005; Del Barrio-Galán et al., 2011; Martínez-Lapuente et al., 2018; Giménez et al., 2019; Unterkofler et al., 2020). However, many of these studies focus on specific polysaccharide families (e.g., mannoproteins) and do not present a holistic overview of the compositional changes occurring to these sugars during sparkling wine production and aging (Martínez-Lapuente et al., 2013, 2015; Martínez et al., 2019; Assunção Bicca et al., 2022). Martínez-Lapuente et al. (2013) evaluated changes to both monosaccharide and polysaccharide components in traditional method sparkling wines produced from five varieties of *V. vinifera* (Verdejo, Viura, Malvasía, Albarín, Godello) over 30 months of aging on *lees* (shown in Table 1). Yeast-derived monosaccharides, namely glucose and mannose, decrease over the aging duration and are associated with low molecular weight polysaccharides, suggesting that these monomers may be consumed in reactions during *lees* aging. However, there does not appear to be a clear trend for glucose or mannose levels between base wine and post-*tirage* (Martínez-Lapuente et al., 2013, 2018). In another study of *V. vinifera* cv. Verdejo by Martínez-Lapuente et al. (2018), lower levels of all sugars were reported compared to those found in Verdejo by an earlier study (Martínez-Lapuente et al., 2013), with the exception of apiose. These differences may be attributed to vintage variation, ripening degrees at harvest, processing practices, or yeast strain selection, although understanding the magnitude of said difference remains unclear. Across both studies, *lees* aged sparkling wines contained mannose as the most abundant sugar, followed by lower levels of glucose, galactose, and arabinose (Martínez-Lapuente et al., 2013, 2018). The implications of trace amounts of sugars derived from grapes and yeast are uncertain in the context of the Maillard reaction during sparkling wine aging and requires further study. Specifically, a study of model systems to identify the potential of Maillard activity at low concentrations, as well as specific “marker” compounds associated with each monosaccharide sugar, would considerably advance knowledge regarding the involvement of unfermentable sugars in the Maillard activity during sparkling wine aging.

**Table 1:** Mean concentrations (mg/L) of monosaccharides in base wines and traditional method sparkling wines aged on lees for wines produced from *Vitis vinifera* varieties.

Sugar	Origin	Mean sugar concentration (mg/L)			Variety	Ref
		Base wine	9 months *lees* aging	30 months *lees* aging		
Apiose	Grape cell walls	n.d.	n.d.	n.d.	Verdejo	^a^
		0.17	0.13	n.a.	Verdejo	^b^
		n.d.	n.d.	n.d.	Albarín	^a^
		n.d.	n.d.	n.d.	Viura	^a^
		n.d.	n.d.	n.d.	Godello	^a^
		n.d.	n.d.	n.d.	Malvasía	^a^
Arabinose	Grape cell walls	33.69	34.89	7.16	Verdejo	^a^
		2.74	2.20	n.a.	Verdejo	^b^
		61.41	22.52	7.03	Albarín	^a^
		42.51	17.58	12.86	Viura	^a^
		24.71	17.51	13.24	Godello	^a^
		31.83	32.92	15.80	Malvasía	^a^
Fucose	Grape cell walls	0.79	1.79	0.54	Verdejo	^a^
		0.17	0.15	n.a.	Verdejo	^b^
		3.95	1.10	0.52	Albarín	^a^
		1.91	0.94	1.23	Viura	^a^
		1.58	0.72	0.65	Godello	^a^
		2.32	1.66	0.62	Malvasía	^a^
Galactose	Grape cell walls	61.93	67.52	26.25	Verdejo	^a^
		12.88	9.47	n.a.	Verdejo	^b^
		125.03	43.61	14.12	Albarín	^a^
		55.01	33.34	27.50	Viura	^a^
		26.98	35.43	18.58	Godello	^a^
		59.84	56.81	31.22	Malvasía	^a^
Rhamnose	Grape cell walls	4.09	8.65	1.84	Verdejo	^a^
		1.96	1.25	n.a.	Verdejo	^b^
		18.07	5.10	1.67	Albarín	^a^
		11.95	6.59	5.11	Viura	^a^
		8.96	5.10	3.81	Godello	^a^
		9.64	7.82	2.06	Malvasía	^a^
Glucose	Yeast glucans	49.30	84.76	13.56	Verdejo	^a^
		6.19	4.79	n.a.	Verdejo	^b^
		146.20	109.92	70.00	Albarín	^a^
		90.61	52.44	24.28	Viura	^a^
		61.23	66.09	20.64	Godello	^a^
		87.11	80.54	26.30	Malvasía	^a^
Mannose	Yeast mannoproteins	28.88	67.80	46.19	Verdejo	^a^
		64.67	60.06	n.a.	Verdejo	^a^
		96.62	40.84	23.44	Albarín	^a^
		70.69	102.00	60.75	Viura	^a^
		37.40	49.00	39.00	Godello	^a^
		55.18	61.00	29.27	Malvasía	^a^
		n.a.	100.7	n.a.	Parellada	^c^

a Martínez-Lapuente et al. (2013); wines fermented with selected winery yeast strain (unspecified).

b Martínez-Lapuente et al. (2018); primary and second fermentations with *Saccharomyces cerevisiae* var. *bayanus* IOC 18-2007.

c Nunez et al. (2005); primary and second fermentations with *S. cerevisiae* IFI 473.

##### *Dosage* composition

Following second fermentation and aging, disgorging removes yeast sediment and adjuvants from the bottle, and *dosage* may be added to the wine. Different types of sugar can be utilized in *dosage,* such as cane sugar, beet sugar, dextrose, liquid sugar (sucrose), or rectified concentrated grape must (1:1 glucose: fructose; Kemp et al., 2015, 2017). However, the origins of sucrose vary, e.g., whether it is derived from sugar cane or sugar beet, thus affecting levels of trace contaminants and volatile compounds derived from sugar refinement processes (Urbanus et al., 2014; Wilson et al., 2022). The type of sugar (glucose, fructose, sucrose), as well as the concentration used in *dosage*, imparts a unique sensory profile on the finished wine (McMahon et al., 2017). To the best of our knowledge, there is no existing research investigating the impact of *dosage* sugar type on Maillard reactions during sparkling wine and aging.

The *dosage* solution accounts for lost volume post-disgorging and is comprised of wine with an optional sugar addition. The wine may be the corresponding sparkling base wine, a different wine type/style, or may occasionally include additives such as liquor (e.g., brandy). The composition of the wine base for the *dosage* solution influences the formation of volatile aroma compounds (Kemp et al., 2017), although implications on the Maillard reaction remain unknown. In future research related to the Maillard reaction in sparkling wine, special attention to the composition of Maillard-associated products in the base solution for *dosage* is highly relevant, particularly in circumstances where fortified wines or spirits are utilized. Indeed, determining the effect of *dosage* composition on the Maillard reaction necessitates an understanding of Maillard-associated compounds which may be directly introduced to the wine in the *dosage* prior to aging.

##### Sugar structure and Maillard activity

Sugar structure affects Maillard reactivity, with pentose sugars (e.g., ribose, xylose, arabinose) reacting more readily compared to hexose sugars (e.g., fructose, glucose), and monosaccharides more readily than disaccharide sugars (e.g., maltose, sucrose; Ames, 1990). Sugar type is also reported to influence the number of distinct Maillard reaction-associated products, where ribose > arabinose > fructose ≈ xylose > galactose > glucose (Hemmler et al., 2018), although this differs from the reported influence of sugar type on browning, where xylose > arabinose > glucose > maltose > fructose (Kwak and Lim, 2004). This discrepancy is likely attributable to differences in the reaction pathway, as some sugars may produce a greater number of distinct intermediate species or breakdown products but decrease the formation of brown pigmented melanoidin compounds.

#### Amino acids

##### Yeast strain

The selection of yeast strain for primary and second fermentation is a critical factor in determining the composition of free and total amino acids in wine which may go on to participate in Maillard activity during prolonged aging. Amino acids may be present in either a free form or as bound components of peptides or proteins. The amino acid composition of sparkling base wine is due to both grape and yeast contributions and consists primarily of proline (Pro; ~30–85%), with alanine (Ala), glutamic acid (Glu), arginine (Arg), γ-aminobutyric acid (GABA), glutamic acid (Glu), lysine (Lys), phenylalanine (Phe), and glutamine (Gln) the next most abundant (Lehtonen, 1996; Moreno-Arribas et al., 1998; Martínez-Rodríguez et al., 2002; Sartor et al., 2021). Although many researchers have studied the contributions of yeast strain to wine qualities (e.g., flavor, stability, chemical composition, foam stability, etc.), a greater understanding of the how yeast strain, autolysis, and amino acid reactivity influence the Maillard reaction during sparkling wine aging conditions is necessary.

Following primary fermentation, *tirage* (combination of yeast, sugar, nutrients, and adjuvant) is added to initiate the second alcoholic fermentation. After the second fermentation, flocculated yeast *lees* aged under pressurized conditions (approximately 6 atm at 20°C) undergo autolysis-driven membrane disorganization, prompting the release of volatile compounds, proteins, peptides, and amino acids into the wine matrix, rendering these available for Maillard reactions (Alexandre and Guilloux-Benatier, 2006; Tudela et al., 2012; Riera, 2016). Thus, yeast strain selection for the second fermentation requires particular consideration for autolytic and flocculation capacity, ethanol tolerance, low-temperature suitability, metabolite production, and enzymatic activity (Torresi et al., 2011; Martínez-García et al., 2017).

Factors such as aging duration, grape variety, yeast, region, and climate can influence amino acid levels in both base and aged sparkling wines (Moreno-Arribas et al., 1998; Martínez-Rodríguez et al., 2002; Hidalgo et al., 2004; Torresi et al., 2011). Several researchers have evaluated the influence of grape variety during aging on yeast *lees* after second fermentation in bottle. In a study by Moreno-Arribas et al. (1998), the influence of grape variety on sparkling wine amino acid profiles during second fermentation and aging was assessed for four single varietal base wines (Macabeo, Xarel.lo, Parellada and Chardonnay) upon inoculation with *S. cerevisiae* var. *bayanus* (strain unspecified). The authors identified that all varieties except for Chardonnay showed a decrease in free and total amino acid content after 9 months of aging on yeast *lees*, while Chardonnay wines showed levels similar to its respective base wine. Moreno-Arribas et al. (1998) also identified that after extended aging on yeast *lees* (18 months for Macabeo, Parellada, and Chardonnay varieties and 24 months for Xarel.lo sparkling wines), amino acid levels decreased, and dropped to levels below those found in corresponding base wines after 31 months of aging. Similarly, Martínez-Rodríguez et al. (2002) reported that the concentration of amino acids is impacted by the yeast strain used in second fermentation, as well as aging duration on *lees*. Yeast strains *S. cerevisiae* var. *bayanus EC-1118* (Lallemand) and two *S. cerevisiae* strains, *IFI-473* and *IFI-475* (Instituto de Fermentaciones Industralies), were added with *tirage* to an unspecified variety of base wine and aged for 20, 40, 90, 180, 270 and 365 day intervals, during which time the concentration of free amino acids decreased in all wines, presumably due to assimilation by viable yeasts (Martínez-Rodríguez et al., 2002). Subsequently, the concentration of total free amino acids increased gradually to the final analysis period in this study (365 days), with differences observed between strains, presumed to be due to varying rates of autolysis. This is in agreement with Sartor et al. (2021) who identified that free amino acids show a net increase over an 18 month lees aging period for Chardonnay (*V. vinifera*) and non-traditional grape varieties (Niagara (*V. labrusca*), Manzoni Bianco, Villenave, and Goethe (hybrids)) fermented with *S. cerevisiae* PB2019 yeast. Sartor et al. (2021) observed an intermediate decrease in total amino acids between 9 and 15 months, presumably due to deamination, decarboxylation, or esterification reactions (Feuillat and Charpentier, 1982; Alexandre and Guilloux-Benatier, 2006), although they may also be consumed in Maillard reactions. However, the concentration and type of amino acids consumed by each reaction may vary. Further research is required to elucidate the concentration requirements of amino acids depending on their subsequent reaction pathways during the wine aging process. Sartor et al. (2021) also observed that between 15 and 18 months of aging, many amino acid concentrations subsequently increased, which was attributed to the hydrolysis of amino acids from peptides and/or proteins released during autolysis, suggesting that compounds released from yeast cells will continue to add amino acids to the wine after cell death. Considerations for not only fermentative quality, but also rates of autolysis are critical for understanding the Maillard reaction in sparkling wine.

###### Saccharomyces cerevisiae

*Saccharomyces cerevisiae* yeast strains are most commonly used for winemaking due to their desirable sensory characteristics and high fermentative capacity, with *S. cerevisiae* var. *bayanus* frequently used for the primary and second fermentations (Torresi et al., 2011; Kemp et al., 2015). In Table 2, major free amino acids in Chardonnay base and sparkling wines produced with *S. cerevisiae* are shown as percent composition to facilitate comparisons across studies with the same grape variety. At 9 and 12 or 15-month intervals following the addition of *tirage*, the amino acids Pro and GABA appear relatively stable, while Ala and Lys increase and Gln decreases for both data sets (Moreno-Arribas et al., 1998; Sartor et al., 2021). The relative relationships of Glu, Arg, and Phe are not consistent across studies, indicating that differences in *S. cerevisiae* strain, grape juice and wine chemistry, and production practices may impact the formation and/or consumption of certain amino acids. As with sugars, different amino groups will exhibit varied reaction rates and therefore resulting Maillard products, as discussed later in this section.

**Table 2:** Major free amino acids (% composition) reported in base wines and corresponding traditional method sparkling wines during 9- and 12-months of aging on lees.

Amino acid	Base wine		9 months *lees* aging		12 months *lees* aging	
	Ref^a^	Ref^b^	Ref^a^	Ref^b^	Ref^a^	Ref^b^^,^^*^
Pro	57.21	85.90	55.95	84.25	59.85	84.25
Ala	6.99	0.53	7.33	1.25	7.06	1.13
GABA	5.46	0.33	5.84	0.42	5.30	0.46
Glu	4.27	0.79	2.60	1.25	3.12	0.97
Arg	3.13	2.17	3.67	0.34	3.40	0.60
Lys	2.37	1.65	3.58	2.43	3.25	2.38
Phe	1.36	1.98	1.08	2.92	0.78	2.90
Gln	0.50	4.10	0.08	0.39	0.03	0.28
Cys	n.a.	0.01	n.a.	n.d.	n.a.	n.d.

a Moreno-Arribas et al. (1998); Chardonnay; fermented with *S. cerevisise*, unspecified strain.

b Sartor et al. (2021); Chardonnay; base wine fermented with *S. cerevisiae* PB2019; second alcoholic fermentation with *S. cerevisiae* PB2002.

* 15 months lees aging duration.

###### Non-*Saccharomyces* yeast

Non-*Saccharomyces* yeasts including *Torulaspora delbrueckii, Pichia kluyveri, Lachancea thermotolerans,* and *Candida/Metschnikowia pulcherrima* are increasingly employed in still wine fermentations alongside *S. cerevisiae* and contribute unique metabolites to the wine flavor profile (Jolly et al., 2014). Comparatively limited research has evaluated the application of non-*Saccharomyces* yeast in sparkling wine production than still wines. Existing studies on non-*Saccharomyces* yeast for traditional method sparkling wine production have evaluated *T. delbrueckii, M. pulcherrima, S. pombe* and *Saccharomycodes ludwigii* as either solitary fermenters or in co-inoculation with *S. cerevisiae* (González-Royo et al., 2015; Medina-Trujillo et al., 2016; Canonico et al., 2018; Ivit et al., 2018; Tofalo et al., 2022). In sparkling wines, non-*Saccharomyces* yeast strains may be useful for metabolite production and amino acid composition, although concerns surrounding the use of these strains for second alcoholic fermentation have been raised, particularly due to the production of select metabolites which can negatively influence sensory attributes (Ivit et al., 2018; Ivit and Kemp, 2018; Cotea et al., 2021).

A study of traditional method sparkling wine second fermentations with non-*Saccharomyces* species by Ivit et al. (2018) assessed the use *Saccharomycodes ludwigii* and *Schizosaccharomyces pombe* as single fermenters for white (*V. vinifera* cv. Airén) and red (*V. vinifera* cv. Tempranillo) base wines, compared to a control treatment with *S. cerevisiae*. Following second fermentation and an aging period of 4 months at 12°C, total amino acid content was higher in the non-*Saccharomyces* fermentations compared to the control treatments fermented with *S. cerevisiae*. This was partly attributed to the preferential assimilation of specific amino acids such as aspartic acid, serine, arginine, and tryptophan by *S. cerevisiae* during fermentation, although broader variances in amino acid release are likely explained by differences in the structural composition of yeast species (Ivit et al., 2018). Additionally, this study reported an increase in precursors to biogenic amines in the non-*Saccharomyces* fermentations, which are produced from the decarboxylation of amino acids and linked to microbial activity of lactic acid bacteria in wine (Ivit et al., 2018; Virdis et al., 2021). Recently, Tofalo et al. (2022) investigated second fermentations in traditional method sparkling wines with single or co-inoculations of *S. cerevisiae* (F6789) with *T. delbrueckii* (TB1) or *Starmerella bacillaris* (SB48). The greatest autolytic potential, measured as the amount of free amino acids (expressed as mg leucine/L) at 270 days post-inoculation was identified in sparkling wines produced with co-inoculations of *S. cerevisiae* + *Starm. bacillaris* (128.00 mg leucine/L) and *S. cerevisiae + T. delbruckii* (107.03 mg leucine/L), compared to a lesser amount for *S. cerevisiae* as a single inoculation (53.96 mg leucine/L). This variation was explained by higher protein concentrations in *T. delbruckii* fermentations and rapid cell death coupled with deregulated autophagy for fermentations involving *Starm. bacillaris*. It is of note that single inoculations of *Starm. bacillaris* did not complete the second alcoholic fermentation, while *T. delbruckii* was successful, demonstrating a stronger fermentative capacity. Therefore, second fermentations or co-fermentations with non-*Saccharomyces* yeasts may be an important area of future research due to the higher concentrations of available amino acids and biogenic amines for Maillard activity.

##### Accelerated autolysis

Novel approaches to accelerate the rate of yeast autolysis have recently been examined (Gnoinski et al., 2021a,b). Microwave, ultrasound, and addition of β-glucanase enzyme treatments of a Chardonnay (*V. vinifera*) base wine (pH 3.18, titratable acidity 6.0 g/l, alcohol 11.1% *v/v*) were compared for their ability to disrupt cell membranes of *S. cerevisiae*, prior to addition with the *tirage* solution for second alcoholic fermentation and aging. The aim of these studies was to enhance the availability of autolytic compounds, including amino acids, peptides, and proteins, among others (Gnoinski et al., 2021a,b). In one study, chemical parameters of phenolics, amino acids, proteins and lipids were monitored at 6-, 12-, and 18-months post-tirage during *lees* aging, with microwave and β-glucanase enzyme treatments showing a 10% increase in total free amino acids at 18-months compared to the control (bottle-aged on *lees*) and ultrasound conditions (Gnoinski et al., 2021a). Future research related to the implications of increased amino acid availability on the Maillard reaction during sur-lies aging, and possible impacts on accelerated Maillard chemistry when treating the sparkling wine matrix with high-energy ultrasound or microwave is necessary.

##### Amino acid structure and Maillard reactivity

When comparing amino acid reactivity, lysine is reported to have the greatest effect on browning, while cysteine contributes the least color (Ames, 1990), although color formation does not necessarily indicate reactivity at all stages of the reaction. In a study of un-buffered model solutions (un-buffered pH range ~2.2–7.0; 100°C; 10 h) with equimolar (0.1 M) combinations of amino acids (glycine, isoleucine, lysine, and cysteine) and ribose sugar, amino acid reactivity was reportedly lysine > cysteine > isoleucine ≈ glycine (Hemmler et al., 2018). Cysteine produced more than 400 distinct Maillard reaction products, despite it being considered a relatively unreactive amino acid in the Maillard reaction (Hemmler et al., 2018), although it has been studied in the context of the Maillard reaction in model sparkling wines (Pripis-Nicolau et al., 2000; Marchand et al., 2011). Cysteine is reported to be found in red wine at concentrations of approximately 1–6 mg/l (Pripis-Nicolau et al., 2001), although it was reported to be below 0.1 mg/l in traditional method sparkling wine aged on *lees* (63% chardonnay, 37% pinot noir; fermented with *S. cerevisiae* IOC 18-2007; Gnoinski et al., 2021a).

Importantly, the rate and relative reactivity of amino acids will be impacted by the reacting sugar type and physicochemical conditions of the system, suggesting that although approximations regarding amino acid reactivity can be made, relevant processing conditions will impact these outcomes (Ames, 1990; Nursten, 2005; Hemmler et al., 2018).

### Metal ion content

#### Metals in sparkling wine

Metals are implicated in enhancing Maillard reactivity, as discussed in the subsequent section. To the best of our knowledge, the potential role of metal ions catalyzing or influencing the Maillard reaction in sparkling wine has not been investigated. Further, limited literature has evaluated the metal composition of sparkling wines. Thus, an understanding of the metal levels present in sparkling wines is necessary for informing future study on the Maillard reaction during aging.

Metals in wine originate from various natural and human-driven sources such as grape variety, soil, water, climate, pollution, processing equipment/storage vessels during winemaking, or by deliberate addition, such as the use of copper-based pesticides for treating downy mildew fungal disease in the vineyard, or the use of other fertilizers and pesticides in viticultural practices (Tariba, 2011). During *V. vinifera* growth and development, the uptake and regulation of various nutrients has downstream implications on the resulting wine quality. Grapevines require 16 essential minerals, including macronutrients (N, P, K, Ca, S, Mg) and micronutrients (Zn, B, Fe, Mn, Mo, Ni, Cl, Cu, Fe, Co) which facilitate essential enzymatic, protein, mitochondrial and chloroplast functions (Migeon et al., 2010). Heavy metals including Pb, Hg, Cu, Ag, Cr, and As are found at higher levels in contaminated soils as a result of agricultural practices or environmental pollution (Tariba, 2011). In winemaking, select metal ions are utilized by yeast as essential nutrients during fermentation, where P, S, K, and Mg are required at higher (millimolar) concentrations, while Na, Ca, Fe, Co, Zn, Mo, Cu, Mn, Ni, and Se are utilized at trace (micromolar or less) levels (Walker, 2004).

The metal composition of sparkling wine has been the subject of limited studies, and has primarily been investigated in the context of authenticity evaluations to discriminate wines by growing region (Greenough et al., 1997, 2005; Taylor et al., 2003; Jos et al., 2004; Vinciguerra et al., 2016; Yamashita et al., 2019; Rodrigues et al., 2020). In 2022, comparison of metal composition in 73 Canadian sparkling wines from the Niagara Peninsula according to production method and style reported higher levels of Cr, Ni and Sr. and lower levels of B in tank-fermented Charmat wines compared to traditional method sparkling wines, as well as lower levels of Cu and higher levels of K in rosé style sparkling wines (Charnock et al., 2022). The authors associated increased Cr, Ni and Sr. levels in Charmat wines to stainless steel contact during production in pressurized vessels, while higher K in rosé wines were associated with extended maceration (Charnock et al., 2022). Cr is a Group II metal, which are known to facilitate greater browning potential in model Maillard reaction systems (Rizzi, 2008; Ramonaityte et al., 2009; Wang et al., 2013). However, the implications of Cr on Maillard activity, especially at trace levels identified in this study (10–43 μg/L), are currently unclear (Kennedy and Knill, 2003).

A recent study on metals in sparkling wines tested Cava during various production stages (must, wine, sparkling wine) to evaluate the influence on metal composition, and identified patterns related to the concentrations of K, Cu, Ca, S, and Mg, which were attributed to the use of bentonite as a stabilizing agent during winemaking (Granell et al., 2022). This was similar to Cellier et al. (2022), who evaluated metals during the production process of Champagne and identified links between compositional differences in the must and bedrock mineral content, while additives, sugar, yeast, and bentonite did not majorly impact elemental levels. Nevertheless, the timing, concentration, and type of additives may also play an important role regarding their impact on wine mineral composition and thus their potential influence on the Maillard reaction.

#### Role of metals in the Maillard reaction

Metals form complexes with organic ligands including amino acids, peptides, and proteins, and may also interact with intermediate Maillard species in a wide pH range (Nashalian, 2016). Conversely, sugars are unlikely to complex with metal ions in neutral or aqueous solutions due to their low stability under these conditions (Gyurcsik and Nagy, 2000). Amino acids coordinate with metals *via* two potential binding sites in amine and carboxylate moieties (O- or N-chelation), or by side chain interactions, which vary according to amino acid structure (Shimazaki et al., 2009). Depending on the metal, the strength of covalent bonding varies with alkali metals Mg and Ca forming weak complexes, while transition metals such as Cu and Zn can form stronger bonds (Shimazaki et al., 2009). When metals are bound to amino acids, the α-CHR group of the amino acid has enhanced acidity, thus enabling aldol reactions with small aldehydes, producing hydroxymethyl-derivatives in a process known as the Akabori transformation (Nashalian, 2016). Trace elements have also been implicated in driving Maillard activity by acting as a Lewis acid catalyst, or an electron pair acceptor, to activate the carbonyl group for nucleophilic attack by the amino group of amino acids, peptides or proteins (Kobayashi and Manabe, 2002; Omari et al., 2021).

Due to this enhanced reactivity, metal ions accelerate the rate of browning in thermally processed Maillard reaction systems (Rizzi, 2008; Ramonaityte et al., 2009; Wang et al., 2013; Omari et al., 2021), although their role in early and intermediate stages and in low-temperature conditions remains poorly characterized (Kennedy and Knill, 2003). The most widely examined role of metal ions in the Maillard reaction is their catalytic effect on brown pigment development associated with melanoidin production in advanced reactions (Rizzi, 2008; Ramonaityte et al., 2009; Wang et al., 2013). Rizzi (2008) demonstrated that cationic species influenced browning at 100°C and pH 7.2, with Group I metals Li, Na, K, Rb and Cs showing a lesser degree of browning compared to Group II metals Ca and Mg. Similarly, Ramonaityte et al. (2009) showed that divalent metal ions of Fe, Cu and Zn accelerate browning in a lactose/glycine model system under reflux. In a different study, a model system with phytic acid showed a catalytic order of Fe > Mg > Ca, according to browning development (Wang et al., 2013). In beer brewing, increasing Mg concentration accelerates Maillard activity based on the formation of brown pigments and high molecular weight polymers (Omari et al., 2021). Although the formation of advanced reaction products and brown pigments is much less relevant in sparkling wine, metal ions are thus likely to drive reactivity in earlier reaction stages, which may be important under mild Maillard conditions. Furthermore, metal ions also accelerate the formation of Amadori compounds in a phosphate buffer solution (Ase et al., 1996), although they may also oxidize these intermediate compounds, catalyze further interactions with Amadori derivatives, and/or form complexes with both primary reactants and reaction products (Ramonaityte et al., 2009).

### Pressure

High hydrostatic pressure (> 5000 atm) decreases the maximum concentration of volatile Maillard reaction products (Bristow and Isaacs, 1999) and decrease the rate of Amadori product formation (Ma et al., 2017). However, studies related to the influence of pressure on the Maillard reaction evaluate pressures much higher than that experienced by sparkling wine (6 atm) and are thus of questionable application. Studies in model sparkling wine systems (pH 3–4; 15 ± 3°C) at pressures relevant to sparkling wine are necessary to determine the influence of pressure on the Maillard reaction in sparkling wines during aging.

### Influence of SO_2_

Sulfur containing compounds including amino acids and SO_2_ have been implicated as inhibitors of Maillard activity (Ames, 1990; Friedman and Molnar-Perl, 1990; Finot, 2005; Nashalian, 2016). While SO_2_ is commonly added during wine production for antioxidant and antimicrobial protection, free SO_2_ may bind to reducing sugars and intermediate Maillard reaction products including carbonyls and dicarbonyls, rendering them inaccessible for Maillard interactions (Ames, 1990). However, in a recent study on the effects of SO_2_ on the Maillard reaction in dried apricots during low-temperature storage, increased SO_2_ levels were correlated to an increase in fructose content (*r* = 0.766), potentially due to the degradation of sucrose to glucose and fructose by SO_2_, invertase activity and/or the conversion of sorbitol to fructose by sorbitol dehydrogenase (Hamzaoğlu et al., 2018).

While research on inhibitory and bleaching effects of sulfur dioxide on advanced browning during the Maillard reaction has been extensively reported (Hodge and Rist, 1953; Wedzicha, 1984; Ames, 1990), further research is necessary concerning the effects on intermediate reaction products, particularly in low-temperature and low pH environments. Notably, sulfur containing Maillard reaction products and heterocycles are often important contributors to aroma in foods and beverages due to low sensory perception thresholds, which range from μg/kg to mg/kg levels (Cerny, 2008).

### Water activity

Water activity (a_w_) is a measure of the residual moisture content, and the Maillard reaction occurs most readily in foods that have low (0.5–0.8) a_w_ values, meaning they are dried or have intermediate moisture levels (Ames, 1990). The presence of non-water volatiles influences a_w_, and in wines, compounds including ethanol, sugars, and other solutes (glycerol, acids, volatile compounds) affect this value (Allan et al., 2019). Based on a sample of 23 commercial sparkling wines, the a_w_ was determined to be between 0.938 and 0.959, with an average of 0.948 a_w_ (Allan et al., 2019), indicating that Maillard reactivity is non-optimal in a_w_ conditions of sparkling wine.

## Maillard reaction-associated products in sparkling wine

### Model wine conditions

Limited research has investigated the Maillard reaction in model conditions, and to the best of our knowledge, there are no existing studies evaluating sparkling wine-specific model conditions which account for CO_2_, pressure, and SO_2_ conditions. Since SO_2_ can inhibit Maillard activity (Ames, 1990; Friedman and Molnar-Perl, 1990; Finot, 2005; Nashalian, 2016), its exclusion is likely beneficial for studying the formation of reaction products under model conditions. However, as previously discussed, the influence of pressure and CO_2_ on the Maillard reaction is uncertain, and future evaluation of their influence on model systems is essential.

One study investigating the Maillard reaction in model wine was carried out by Pripis-Nicolau et al. (2000). Model wines were prepared to closely replicate the physicochemical environment of sparkling wine including low pH (pH 3.5) and low-temperature hydroalcoholic conditions (10 and 25°C conditions; 12% *v/v* alcohol; 20 mmol/l stoichiometric combinations of amino acids (arginine, phenylalanine, lysine, cysteine, methionine, proline, valine, leucine, isoleucine, glycine, serine, alanine, γ-aminobutyric acid, glutamic acid) and α-dicarbonyls (glyoxal, methylglyoxal, diacetyl, pentan-2,3-dione, acetoin, acetol)). The reactions were studied over a 4 week period and results indicated that the sulfur-containing amino acids, in particular cysteine, were associated with the formation of intermediate Maillard heterocycle compounds including pyrazines, methylpyrazines, methylthiazoles, acetylthiazoles, acetylthiazolines, acetylthiazolidines, trimethyloxazole, and dimethylethyloxazoles in both 10 and 25°C temperature conditions (Pripis-Nicolau et al., 2000). The authors reported the formation of 2-acethylthiazole and 2-acetyl-2-thiazoline to occur rapidly in these model wine conditions (<3 h; Pripis-Nicolau et al., 2000). Additionally, Strecker degradation products of 2-methylpropanal, 3-methylbutanal, 2-methylbutanal, and phenylacetaldehyde were also identified in model conditions containing valine, leucine, isoleucine, and phenylalanine amino acids, respectively. Further, benzaldehyde was also identified in reactions containing phenylalanine. However, the concentrations of these Strecker aldehydes were not reported in model systems (Pripis-Nicolau et al., 2000).

Despite its low concentration, cysteine has been suggested to be an important amino acid in Maillard reactions in sparkling wine due to its involvement in the formation of heterocyclic aroma compounds. This is assumed to be linked to the electron density in the primary amine functional group, which is highly reactive with the electrophilic carbonyl moiety of a reducing sugar (Marchand et al., 2000). Marchand et al. (2000) also evaluated the role of cysteine in the formation of Maillard reaction products in real wine and model wine conditions (20°C, 12% *v/v* ethanol, pH 3.5), and also identified a correlation between cysteine and thiophene-2-thiol levels in Champagne wines, with levels of 1 μg/L, above the sensory detection threshold in water (~0.8 μg/L) indicating an OAV of 1.4 (Marchand et al., 2000). In more recent model sparkling wine study, Marchand et al. (2011) investigated the reaction between cysteine and diacetyl under wine-like conditions (20 ± 2°C, 12% *v/v* ethanol, pH 3.5). Diacetyl is a α-dicarbonyl, which readily reacts with amino acids *via* Strecker degradation. Identified reaction products included 2-methylthiazole, 2-methyl-2-thiazoline, 2-methylthiazolidine, 2,4,5-trimethyloxazole, 2-acetyl-2-methyl-3-thiazoline, 2-acetyl-2-methylthizolidine, 2-(1-hydroxyethyl)-2,4,5-trimethyloxazoline, and tetramethylpyrazine, along with carbonyl degradation products of acetic acid and acetoin (Marchand et al., 2011). However, as these studies were carried out in model wine, it remains unclear if all compounds are identifiable, formed, or present in finished and/or aged sparkling wines, especially with little to no cysteine in some aged sparkling wines.

### Finished sparkling wines

Maillard reaction-associated products identified in finished sparkling wines in literature are reported in Table 3. Tominaga et al. (2003b) identified thiols (benzenemethanethiol, 2-furanmethanethiol, and ethyl 3-mercaptopropionate) in aged Champagne wines (ranging from 0–27 years of total bottle aging time including pre-and post-disgorging time). This is in agreement with Marchand et al. (2000), who identified four heterocyclic compounds (thiazole, 2-acetylthiazole, trimethyloxazole, and thiophene-2-thiol) in seven Champagne wines, and suggested they were derived from the Maillard reaction involving cysteine. Notably, the authors identified similar levels of thiazole in both Champagne and fortified wines, contributing “popcorn” and “peanut” aromas, although all measured values were below the odor perception threshold of 38 μg/L. Additionally, thiophene-2-thiol was reported to have a mean concentration of 1 μg/L in Champagnes and an odor perception threshold of 0.8 μg/L, indicating an OAV of 1.4, similar to the OAV of 1.7 for thiophene-2-thiol in Pomerol and Saint-Emillion still red wines. In another study, Keim et al. (2002) evaluated the *N*-heterocyclic compounds, 2,4,5-trimethyloxazole, 4-methylthiazole, 2,4-dimethylthiazole, and 2-acetyl-2-thiazoline, in fortified, still, botrytized, and sparkling wines. In fortified wines, 2,4,5-trimethyloxazole was found at levels between 0.3 and 1.3 mg/l but was not detected in sparkling wines. Conversely, 4-methylthiazole in sparkling wines was found between 0 and 0.4 mg/l, although not detected in fortified wines. Differences between 2,4,5-trimethylthiazole and 4-methylthiazole levels in fortified and sparkling wines were suggested to be linked to alcohol content (fortification) for 2,4,5-trimethyloxazole, and sparkling wines aging in the presence of yeast *lees* for 2-acetylthiazole (Keim et al., 2002). Other Maillard reaction-associated compounds including 2,4-dimethylthiazole, 2-acetylthiazole, and 2-acetyl-2-thiazole were identified in sparkling wines at ranges of 0–0.3, 0–0.4, and 0–0.2 mg/l, respectively.

**Table 3:** Maillard reaction-associated products identified in sparkling wines.

Compound	Analytical method	Sparkling wine	Reported concentration in sparkling wines	Aroma descriptor	Odor threshold
2-furanmethanethiol	GC–MS	Champagne	2–5500 ng/L^a^	Roasted coffee^b^	0.4 ng/L^b^^,^^†^
ethyl 2-mercaptopropionate	GC–MS	Champagne	50–800 ng/L^a^	Tropical fruit^c^	500 ng/L^d^
ethyl 3-mercaptopropionate	GC–MS	Champagne	40–12000 ng/L^a^	Foxy^e^	200 ng/L^e^
benzenemethanethiol	GC–MS	Champagne	10–400 ng/L^a^	Smoke, burnt wood^f^	0.3 ng/L^f^
thiazole	GC-NPD	Champagne	0-23 μg/L^g^	Popcorn, peanut^g^	38 μg/L^g^^,^^*^
trimethyloxazole	GC-NPD	Champagne	0–5 μg/L^g^	Very ripe fruit^g^	17 μg/L^g^^,^^*^
2-acetylthiazole	GC-NPD	Champagne	0–3 μg/L^g^	Roasted hazelnut^g^	3 μg/L^g^^,^^*^
	GC–MS	Champagne	0-0.4 mg/L^h^		
thiophene-2-thiol	GC-NPD	Champagne	0–4 μg/L^g^	Roasted coffee, burnt^g^	0.8 μg/L^g^^,^^*^
4-methylthiazole	GC–MS	Champagne	0–0.4 μg/L^h^	Green hazelnut^g^	55 μg/L^g^
2,4-dimethylthiazole	GC-NPD	Champagne	0–0.3 mg/L^h^	Peanuts, roasted, oxidized beer, roasted red meat, coffee^g^^,^^h^	n.a.
2-acetyl-2-thiazoline	GC–MS	Champagne	0–0.2 mg/L^h^	Roasted hazelnut^i^	<5 μg/L^i^
5-HMF	UHPLC-DAD	Cava	0.4–1.7 mg/L^j^	n.a.	n.a.
2-methylpropanal	GC–MS/MS	Traditional method	7–23 μg/L^K^	Malty^l^	6 μg/L^l^^,^^†^
2-methylbutanal	GC–MS/MS	Traditional method	2–35 μg/L^K^	Malty^l^	16 μg/L^l^^,^^†^
methional	GC–MS/MS	Traditional method	0.1–3.8 μg/L^K^	Cooked potato^m^	0.5 μg/L^m^^,^^†^
2-phenylacetaldehyde	GC–MS/MS	Traditional method	2–11 μg/L^k^	Honey, floral^l^	1 μg/L^l^^,^^†^

n.a., not available; GC–MS, gas chromatography–mass spectrometry; GC-NPD, gas chromatography-nitrogen phosphorous detector; UHPLC-DAD, ultra-high-performance liquid chromatography-diode array detector; GC–MS/MS, gas chromatography tandem mass spectrometry.

a Tominaga et al. (2003b).

b Tominaga et al. (2000).

c Meng et al. (2014).

d Blanchard (2000).

e Kolor (1983).

f Tominaga et al. (2003a).

g Marchand et al. (2000).

h Keim et al. (2002).

i Jackson (1996a).

j Serra-Cayuela et al. (2013a).

k Sawyer et al. (2022).

l Culleré et al. (2007).

m Escudero et al. (2000).

† Odor threshold reported in model hydroalcoholic solution.

* Odor threshold.

As previously discussed, low pH Maillard reaction conditions favor the formation of furfural intermediates, although they are also produced during acid-catalyzed sugar degradation reactions. This is consistent with the presence of 5-HMF identified in Cava sparkling wine (Serra-Cayuela et al., 2013a).

Strecker degradation products 2-methylpropanal, 2-methylbutanal, methional and phenylacetaldehyde have been identified in aged sparkling wines produced from Chardonnay and Pinot noir at concentration ranges of 7–23, 2–35, 0.1–3.8, and 2–11 μg/L, respectively (Sawyer et al., 2022). Concentrations were monitored at 6-, 12- and 24-months bottle aging, and in all cases, the highest levels were reported at 24-months aging, where all concentrations exceeded the reported odor thresholds. Aroma descriptors for these Strecker aldehydes include malty, cooked potato, honey, and floral aromas, which is consistent with higher sensory scores for honey, nutty, and autolytic aromas at 24-month compared to 12-month aged wines (Sawyer et al., 2022).

While many other Maillard reaction-associated compounds have been identified at concentrations above their odor perception detection threshold, further sensory study is required to evaluate the perceived influence of these compounds on the aroma profile and consumer preference of sparkling wines.

## Analytical methods for Maillard product determination

The separation and quantitative determination of Maillard reaction compounds have been carried out using a range of targeted chromatographic techniques. As previously mentioned, the degree of browning index measured by UV–Vis at 420 nm is commonly used to monitor Maillard activity and reaction kinetics (Lingnert and Eriksson, 1980; Laroque et al., 2008), although in wine, oxidative browning interferes with this measurement.

Generally, targeted techniques including Gas Chromatography (GC) and High-Performance Liquid Chromatography (HPLC) are utilized for the analysis of known or predicted Maillard reaction products (Hemmler et al., 2017; Xing, 2021). When coupled to mass spectrometry (MS), GC–MS may be used for the determination of volatile and semi-volatile chemical species while HPLC-MS is suitable for non-volatile components and has relatively low costs, and low detection limits (μg/L) and is broadly suitable for flavor-relevant compounds.

The earliest study concerning the Maillard reaction in sparkling wine was carried out by Pripis-Nicolau et al. (2000) and quantified Maillard reaction products by GC – Flame Photometric Detector (FPD), GC – Nitrogen-Phosphorous Detector (NPD), GC – Flame Ionization Detector (FID), and GC–MS (compounds identified in sparkling wines reported Table 3). Later, Keim et al. (2002) published a method for the analyses of *N*-heterocycle compounds in sparkling wines by GC-NPD and GC–MS. In 2013, a consolidated method for the determination of Maillard-associated chemical compounds was published by Burin et al. (2013), which utilized Solid-Phase Microextraction (SPME) GC–MS as a solvent-free and extraction-free alternative to other GC methodologies. This technique improved upon previous methods by reducing sample manipulation and enhancing compound specificity, demonstrating broad applicability for heterocycles of interest in sparkling wine. Additionally, Serra-Cayuela et al. (2013a) utilized Ultra-High-Performance Liquid Chromatography – Diode Array Detector (UHPLC-DAD) for the quantification of 5-HMF.

Equally, non-targeted analytical techniques aim to investigate the entire sample, thus capturing complexities that may be overlooked or inaccessible to targeted methods (Forcisi et al., 2013; Hemmler et al., 2017; Xing, 2021). More recently, high-resolution mass spectrometry methods have been investigated as non-targeted analytical techniques for understanding and controlling the Maillard reaction (Nashalian et al., 2016). These methods may be used to accurately determine the elemental composition and elucidate the structure of compounds in a complex matrix.

Diagnostic techniques that have been used for understanding the Maillard reaction cascade and characterizing the formation of intermediate species include Electrospray Ionization Quadrupole Time of Flight Mass Spectrometry (ESI-qTOF-MS) (Golon and Kuhnert, 2013; Golon et al., 2014; Nashalian et al., 2016; Omari et al., 2021), Tandem Mass Spectrometry (MS/MS) (Xing et al., 2020), Ultrahigh-Resolution Ion Cyclotron Resonance Mass Spectrometry (FT-ICR-MS) (Golon et al., 2014; Jeandet et al., 2015; Hemmler et al., 2017) and Nuclear Magnetic Resonance (NMR) (Jeandet et al., 2015). More recently, Jeandet et al. (2015) and Serra-Cayuela et al. (2013b) employed one-dimensional ^1^H NMR to confirm the presence of 5-HMF in Champagne and Cava sparkling wines, although quantitative NMR was not employed. Jeandet et al. (2015) studied a 170-year-old champagne found in a shipwreck in the Baltic Sea, which showed a relative peak area of 0.4 for 5-HMF while no HMF was detected for three more recent Champagne wines (1980, 1955, and 2011 Veuve Clicquot, unspecified *lees* aging durations or *dosage* composition). Quantitative ^1^H NMR methods would significantly improve the application and scope of this study in relation to Maillard product determination in wines, due to the unambiguous structural confirmation of small molecules, rapid sample throughput, and minimal volume requirements. To the best of our knowledge, quantitative NMR has not been applied to sparkling wine studies, although it was recently evaluated as a technique for still wine authenticity with promising results, particularly when paired with traditional wine chemistry analysis approaches (Gougeon et al., 2019). However, high detection limits are the primary drawback of this technique in an application to wine studies. Mechanochemistry has also been investigated in the context of food science as a non-targeted, solvent-free and rapid analysis technique, although this field is still in its infancy (Xing, 2021).

## Conclusion

Due to the complexity of the Maillard reaction and the host of compounds that can result from a single sugar and amino acid interaction, it remains one of the most challenging topics in food chemistry. Evidence of Maillard reaction-associated compounds in aged traditional method sparkling wine underlines the need for further research in this area, especially due to the potential sensorial contributions of many Maillard-associated compounds. While the Maillard reaction in sparkling wine is inhibited by low pH, low temperature, SO_2_ presence, and high a_w_, these are countered by a long reaction time (months to years of aging on *lees*), diverse sugar and amino acid composition, low pressure, and metal ion content. Due to low pH and low temperature conditions as well as acid-catalyzed sugar degradation reactions, the Maillard reaction in sparkling wine likely proceeds to the intermediate stage and favors the formation of furfural compounds (e.g., 5-HMF), which may be useful chemical markers during aging. Further, the influence of specific Maillard-associated compounds on the aroma of sparkling wines requires further sensory study.

Currently, the specific pathways to the formation of many Maillard intermediates in sparkling wine are unexamined or poorly characterized. Additionally, the influence of residual unfermented sugars, exogenous sugar additions *dosage*, and α-dicarbonyl compounds on the Maillard reaction during sparkling wine aging requires further investigation. Future studies will benefit from model conditions with wine-like temperature conditions rather than heated or accelerated aging strategies, since the development of specific Maillard reaction products is highly dependent on the time and temperature of the system.

Targeted research into the composition of reactant compounds (amino acids and sugars) from different *V. vinifera* or hybrid grape varieties and *S. cerevisiae* and/or non-*Saccharomyces* yeast strains will inform the availability of reaction precursors, and thus potentially direct the preferential use of different grape varieties and/or yeast strains for optimal Maillard potential during aging. Emerging technologies to accelerate the autolysis of yeast following second fermentation also presents an opportunity to increase reaction precursors prior to aging. Additionally, metal ions in wine may drive Maillard activity by acting as a Lewis acid catalyst and require further examination both in terms of composition as well as Maillard implications. Advances in diagnostic and targeted analytical techniques to characterize the composition of Maillard reaction products and interactions during the reaction pathway will be essential to characterizing the Maillard reaction under sparkling wine conditions where browning color changes cannot be reliably used as an indicator of reaction progress. Research on the formation of Maillard-associated products in sparkling wine may reveal strategies to achieve enhanced aroma in the production of traditional method sparkling wines and identify links to the production process.

## Author contributions

HC prepared the manuscript. BK and GP assisted with revisions and additions to the manuscript. All authors contributed to the article and approved the submitted version.

## Funding

This work was funded by the National Sciences and Engineering Research Council of Canada (NSERC) Discovery Grant (RGPIN-2018-04783) to BK. HC gratefully acknowledges the NSERC Canadian Graduate Scholarship – Doctoral (CGS D). This publication was supported by the Brock University Library Open Access Fund.

## Conflict of interest

The authors declare that the research was conducted in the absence of any commercial or financial relationships that could be construed as a potential conflict of interest.

## Publisher’s note

All claims expressed in this article are solely those of the authors and do not necessarily represent those of their affiliated organizations, or those of the publisher, the editors and the reviewers. Any product that may be evaluated in this article, or claim that may be made by its manufacturer, is not guaranteed or endorsed by the publisher.
